# Preliminary image findings of lower limb stress fractures to aid ultrasonographic diagnoses: A systematic review and narrative synthesis

**DOI:** 10.1177/1742271X21995523

**Published:** 2021-03-09

**Authors:** Madeleine Schaper, James Harcus

**Affiliations:** 1 Faculty of Medicine and Health, University of Leeds, Leeds, UK; 2 Leeds Institute of Cardiovascular and Metabolic Medicine, University of Leeds, Leeds, UK

**Keywords:** Sports injury, bone fracture, periosteal thickening, cortical, hypervascularity, ultrasound, Doppler

## Abstract

**Introduction:**

This systematic review investigates which image appearances are most common when diagnosing lower limb stress fractures using ultrasound imaging, with the aim of outlining an image critique guideline for operators to support confident diagnoses.

**Method:**

A comprehensive literature search of medical databases and handsearching was undertaken to identify relevant studies. All studies were critically examined for quality using the CASP critical appraisal tool. Results from eight studies were combined and interpreted using a narrative synthesis.

**Findings:**

A clear outline of common stress fracture appearances using ultrasound were identified in a combined total of 119 participants. Each finding was ranked according to its popularity. Periosteal thickening (78/119) and cortical disruption/irregularity (83/119) were noted in all eight studies. Hypervascularity of the periosteum visualised by colour Doppler imaging (66/119) was reported in six of the eight studies. Soft tissue hypervascularity (13/119), bony callus formation (5/119) and cortical break (22/119) were seen in three studies.

**Conclusions:**

Based on the findings, we offer a guideline of the most significant preliminary image findings to be utilised by operators when examining athletes suspected of having lower limb stress fractures. The results show a gap in research for evaluating changes in appearance depending on the injury severity. Further research into distinguishing stress fractures from pathological involvement may in future reduce reliance on plain film radiography.

## Introduction

### Background

Lower limb stress fractures pose an ongoing challenge for orthopaedic specialists, accounting for approximately 20% of all sports-related injuries.^1^

When a load is applied to a bone, it deforms according to its elastic range and returns to its original shape upon load cessation. When this load exceeds the bone elastic range, commonly seen alongside a sudden activity increase, the bone deformation is not enough to absorb the load-force and can ultimately form microfractures.^2^ Bone formation and bone reabsorption increases with loading activities to restore and repair the skeletal system. Osteocyte apoptosis reabsorbs damaged bone cells, followed by osteoblastic activity for targeted bone remodelling. This cycle of bone reabsorption temporarily weakens the bone, by increasing porosity and decreasing the bone elasticity until full mineralisation of the new tissue.^2^ Therefore, retaining repetitive loading during a stress reaction can promote stress fracture formation due to force repetitively exceeding the bone’s remodelling capacity.

Athletes typically ignore symptoms of pain to prevent training interruption and thereby risk increasing the severity of their injury.^3^ When retaining a repetitive workload without adequate time adaption, an athlete risks long-term muscle fatigue, weakness and reduced shock absorption in the affected bone. Diagnostic imaging of lower limb stress fractures validates the importance of rehabilitation for athletes, which ultimately ensures adequate healing and an overall reduced time-off-sport.^3^

Clinical presentation encompasses localised pain and swelling that typically increases with activity and decreases with rest.^2^ A stress fracture should be suspected if the patient reports a sudden increase in physical activity. However, the clinical presentation can be imprecise, introducing differential diagnoses for lower limb soft tissue injury, including tendinopathy, compartment syndrome and tumours. Medical imaging has the role of identifying a lower limb stress fracture and initiating optimal treatment.^2^

Previous literature has concluded magnetic resonance imaging (MRI) as the ‘gold standard’ imaging modality, with 100% specificity and sensitivity.^4,5^ Nonetheless, with low accessibility and high cost, MRI is unsuitable for many early diagnoses in symptomatic athletes.^4^ Therefore, radiographic plain film imaging is utilised as an initial reference in current clinical management, despite the modality’s low sensitivity (37.02% to 56%) and specificity (88% and 95.45%).^5,6^ Radiographic imaging requires evident callus formation to visualise bony abnormality, typically occurring at three weeks post-symptom onset.^4^ Therefore, radiographic application is limited in early diagnoses, questioning the justification of the radiation exposure in early symptomatic athletes.

Current literature fails to challenge the applicability of plain film radiography. Systematic reviews focus on evaluating a range of imaging modalities on their accuracy when diagnosing stress fractures, without analysing key image findings.

This review advocates the use of ultrasound with its low cost, high accessibility and accuracy.^4^ With MRI as a reference standard, ultrasound imaging occupies a specificity ranging from 76% to 77.27% and a sensitivity between 43% and 86%.^5–7^ We will assess qualitative data extracted from observational case reports and cohort studies to accumulate relevant preliminary image findings for a positive stress fracture diagnosis. The potential of on-site ultrasound imaging may enable faster diagnoses and establish early rehabilitation intervention to reduce the return-to-sport time for symptomatic athletes.^4^

## Methods

### Research aim

The researcher applied the ‘PICO’ model^8^: *Population* as patients symptomatic with a lower limb stress fracture; *Interventions* as ultrasound imaging for diagnosing lower limb stress fractures; *Comparisons* as ultrasound findings from different observational case reports and cohort studies; *Outcomes* of clear ultrasound preliminary imaging findings for successful lower limb stress fracture diagnoses.

Research relied on the documentation of image interpretation by ultrasound operators.

### Search strategy

Databases searched were CINHAL (Cumulative Index to Nursing and Allied Health Literature), PubMed and Ovid Medline. Through the Ovid Medline database, the author combined the University Library's Journals@Ovid, Ovid MEDLINE and Embase (online Appendix B). All databases covered the timeframe from 2009 to January 2020 (online Appendix A).

Key words for database searches were formulated independently using the PICO methodology and combined using the Boolean operators (online Appendix A). Search strings were combined for topics: ‘ultrasound’, ‘stress fracture’, ‘clinical findings’ and adapted for each database. The key term ‘ultrasound’ was mapped to include ‘ultrasonography’ using the Boolean operator ‘OR’ (online Appendix A). All key terms were combined using the ‘AND’ function. Truncation (*) was used to retrieve all words with the same stem to increase the sensitivity of the search results.^10^

The precision of search, the proportion of relevant studies identified by a database search strategy,^9^ was 4.59%.

The hand-searched results, using Google Scholar, were sorted by ‘Best Match’ and filtered since the year 2009, screening 20 studies on pages one and two.

All references were extracted, and duplicates were removed by hand. Literature was kept broad to establish a wider understanding of bone stress appearances using ultrasound.

All searches were conducted and analysed according to the Preferred Reporting Items for Systematic Reviews and Meta-Analysis methodology (PRISMA)^10^ (Figure 3).

### Selection criteria

**Figure 1.**
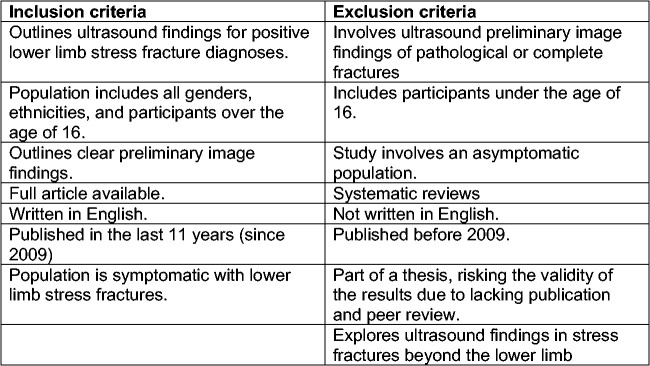
Exclusion and inclusion criteria.

All articles were screened according to inclusion and exclusion criteria (Figure 1). Studies that involve participants under the age of 16 were excluded, due to the varied bone mineral density during childhood.^11^ We also excluded publications before 2009 for a logical timeframe^12^ and included the valuable study by Banal et al.^13^

### Critical appraisal

**Figure 2.**
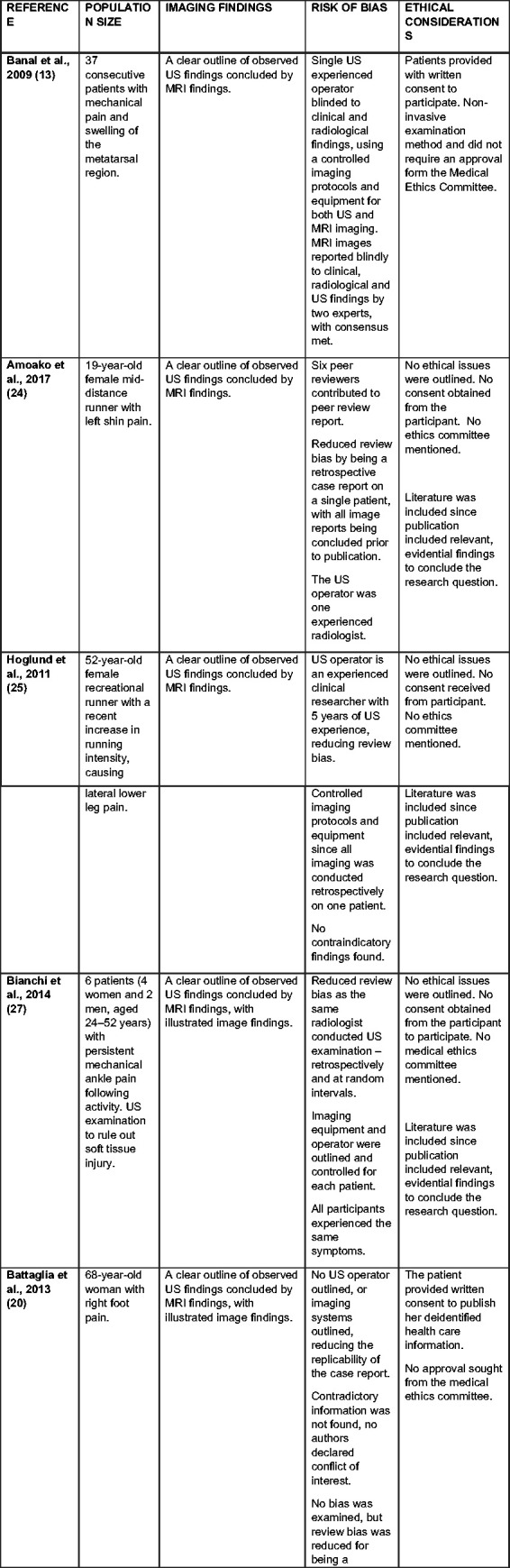

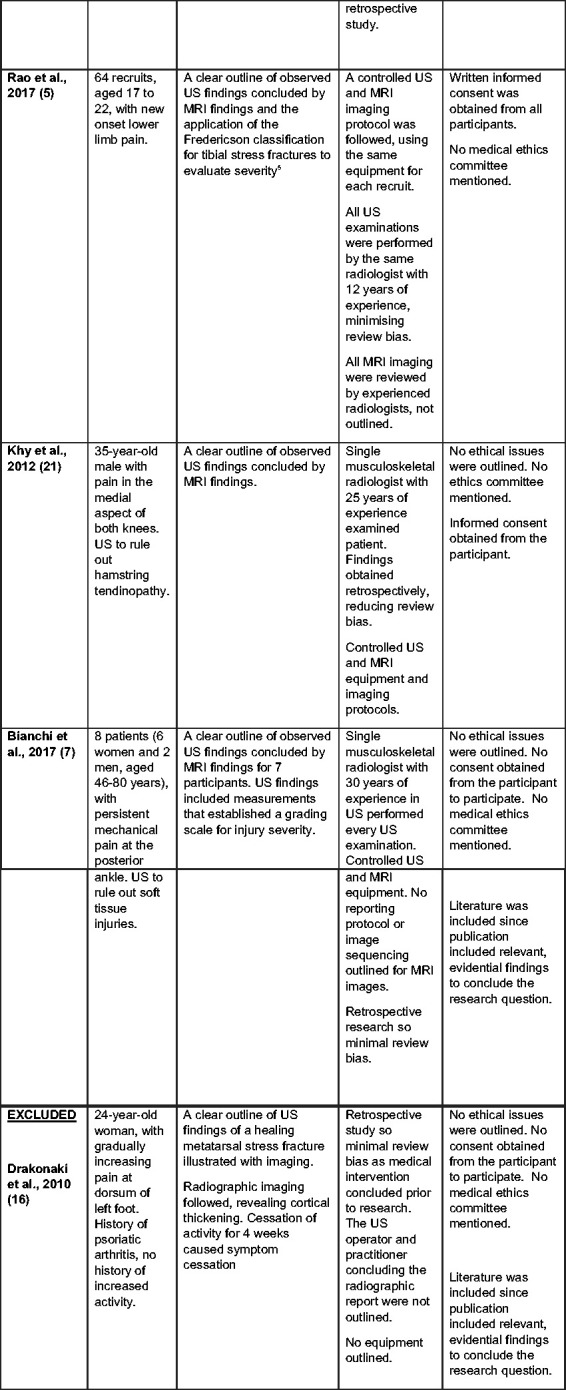
Critical appraisal of all included studies. US: ultrasound.

The authors utilised the Critical Appraisal Skills Programme (CASP) to assess study quality. The CASP Qualitative Checklist was used for all papers to retain a homogeneous approach. The absence of a checklist specifically for case reports reduces the ability to scrutinize literature^14,15^ (Figure 2). 

The process excluded the study Drakonaki et al.^16^ to minimise pathological interference. The participant had psoriatic arthritis, known to reduce bone mineral density.^17^ All included studies considered bias and ethical criteria and provided valuable evidence to answer the research question.

All case reports were written retrospectively to medical intervention; therefore, no approval from the Medical Ethics Committee was required.^18,19^ All studies utilised strictly desensitised information to prevent participant identification. Studies by Banal et al.^13^; Battaglia et al.^20^; Rao et al.^5^ and Khy et al.^21^ obtained written consent from participants to permit the use of desensitised information.

### Data extraction

A table was designed to extract the following information from the studies: author, population size, ultrasound image findings under pre-defined headings.

Due to clinical heterogeneity, a narrative synthesis was conducted to identify any patterns within the data.^22^ The four-step methodology proposed by the Cochrane Consumers and Communication Review Group (CCCRG) was utilised for guidance.^22^ To evaluate significance, the qualitative results were pooled into a super-set of evidence from a combined total of 119 participants.^23^

## Results

### Literature search

The comprehensive search strategy up to 3 January 2020 (online Appendix A) yielded 305 papers: 285 from electronic databases; 20 from handsearching. The electronic database search lowered to a total of 193 papers, after duplicates and publications before 2009 were removed. These 193 papers were screened via the title, removing 153 papers from the search for irrelevancy. Selection criteria excluded 26 studies via abstract screening: systematic reviews (n = 16); no full text availability (n = 5); pathological involvement (n = 2); duplicates within the combined database search (n = 3). The CASP critical appraisal removed one study, for pathological involvement.

**Figure 3.**
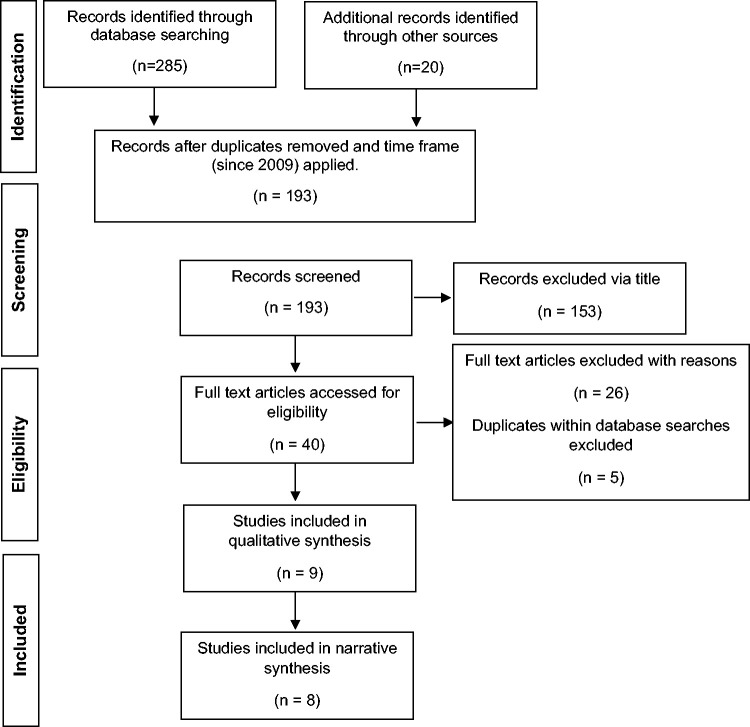
PRISMA flow diagram of study selection process.

The conclusive eligibility checks accumulated eight relevant studies (Figure 3).

### Data extraction

Data from a total of 119 participants were extracted. The terms ‘cortical disruption’ and ‘cortical irregularity’ were grouped due to similar implication. Where studies permitted, each participant’s stress fracture findings were recorded individually for superior accuracy. Nonetheless, the study by Banal et al.,^13^ with 37 participants, failed to outline the findings for each participant. Therefore, each finding recorded was classified as occurring 37 times, thus affecting the overall relationship between the findings. Blank spaces in the table occurred because of a lack of detail in studies.

### Theoretical model

**Figure 4.**
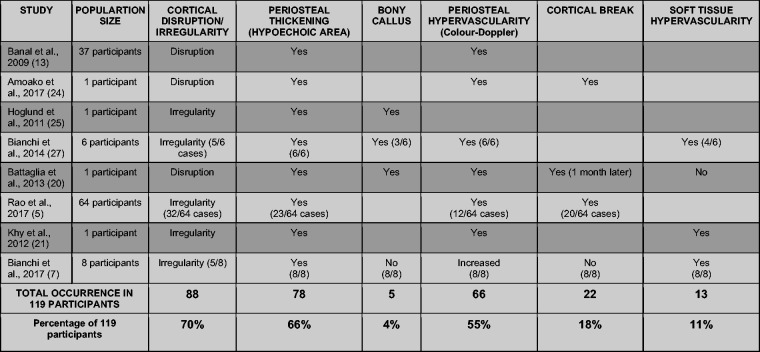
The preliminary image findings observed with total occurrences.

**Figure 5.**
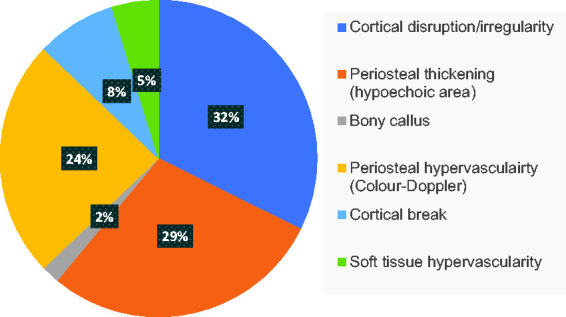
Pie chart to illustrate significance of ultrasound findings in a total of 119 participants across eight studies.

Figures 4 and 5 demonstrate distinct preliminary imaging findings, formulating a theoretical image critique guideline according to relevancy. The specification below can aid operators to make diagnoses confidently and accurately.

1. Is there cortical disruption/irregularity or thickening?
2. Is there periosteal thickening via a hypoechoic area superior to bony cortex?
3. Is there periosteal hypervascularity at the focal area visualised using colour Doppler imaging?
4. Is soft tissue hypervascularity noted at the focal area using colour Doppler imaging?
5. Can a bony callus be visualised?
6. Is there a cortical break seen?

## Discussion

### Narrative synthesis

The population size ranged from 1 to 64 participants per study with an average female percentage of 52%. The variety of research objectives between the eight studies provided the review with generalised image findings for lower limb stress fractures: two studies evaluated metatarsal stress fractures; three studies evaluated tibial stress fractures; one study evaluated fibular and malleoli stress fractures; one study evaluated a calcaneal stress fracture.

Figures 4 and 5 illustrate relevancy in findings, with periosteal thickening (78 participants) and cortical disruption/irregularity over the focal pain area (83 participants) occurring in all eight studies. Hypervascularity of the periosteum visualised on Doppler was also a predominant finding in positive lower limb stress fractures cases, reported in 66 participants in seven studies, thereby seen in 24% of all participants.^7,13,20,21,24,25^ Soft tissue hypervascularity (13 participants^20,21^), bony callus formation (five participants^13,20,25^) and cortical break (22 participants^20,21,24^) were the least common findings. This establishes an informal significance in identifying periosteal thickening, cortical disruption/irregularity over the focal area and periosteal hypervascularity due to an increased likelihood of positive incidence (Figure 5).

This informal ranking follows the pathophysiological development of stress fractures, since bone stress from repetitive deformity initiates a periosteal reaction, visualised as periosteal thickening, the most common finding in this study.^26^ Pathological progression results in osteoblastic activity for targeted bone remodelling, associated with cortical irregularity.^2^ Vascularisation is an integral part of bone remodelling with new blood vessels delivering nutrients and growth factors to osteoblast cells and can be identified using Doppler imaging.^26^ Over time, callus formation increases as more bone tissue is laid down, and therefore bony callus is less common as it is a progression at the latest stage of the stress reaction.^2^

### Strengths and limitations

This systematic review was not registered at PROSPERO. Due to the scarcity of current literature, we utilised case reports and cohort studies despite accumulating low-quality evidence with high clinical heterogeneity^9^ (online Appendix C). The evidence from this review will not change over time, thereby recommendations will not be reversed in the future.^9^ The clinical heterogeneity risks bias in the collated statistics (Figure 5). Nonetheless, this data super-set formed a large population size which increased the credibility of the data analysis.^14^

The clinical heterogeneity is influenced by methodological differences and variable populations sizes.^22^ Rao et al.^5^ had a study population size of 64 military recruits and a methodology that incorporated the use of colour Doppler examination and MRI to follow up ultrasound findings. Conversely, Hoglund et al.,^25^ with a single participant, omitted the utilisation of colour Doppler and MRI. This difference in imaging protocols and population size minimises the comparability and risks contradictory observation.^22^ Additionally, Hoglund et al.,^25^ Bianchi et al.^27^ and Battaglia et al.^20^ observed positive bony callus findings for a total of five participants. However, Bianchi et al.^7^ did not record any occurrences of bony callus formation in all eight participants, thereby contradicting the significance of this finding. Furthermore, they did not rationalise the lack of bony callous findings; however, this finding is associated with late stage diagnoses. Therefore, their eight participants can be assumed to have been imaged prior to this pathological progression.

The discrepancies in the results table questions the relevancy of the evidence to clinical practice. Banal et al.,^13^ Amoako et al.,^24^ Hoglund et al.^25^ and Bianchi et al.^27^ provided similar findings that follow the specifications proposed by this review, suggesting cortical irregularity as a prominent appearance. However, Rao et al.^5^ and Bianchi et al.^7^ reduce the credibility with only a 50% and 62.5% occurrence: 37 participants did not demonstrate cortical irregularity.

Similar discrepancy is seen in the case of periosteal hypervascularity, evident in all six participants in Bianchi et al.’s study^27^ but only documented in 12 of the 64 cases in the one by Rao et al.^5^ Evidence of a cortical break was less frequent, noted in three of the eight studies. Rao et al.^5^ recorded a positive occurrence rate for cortical breaks of 31.3%, whereas Bianchi et al.^7^ had 100% negative occurrence in all their participants.

All studies introduced inevitable measurement bias due to the operator dependency of the qualitative data: each ultrasound operator had an individualistic approach with a lack of practical standardisation.^9^ To maximise internal validity, Banal et al.^13^ utilised blinding, thereby limiting the influence of clinical and radiological presentation on image interpretation.

Selection bias was high in studies recruiting patients with positive stress fracture diagnoses, thereby reducing the ability to investigate differential diagnoses using ultrasound. Amoako et al.^24^ utilised purposeful sampling to document a positive lower limb stress fracture case from a 19-year old patient.^23^ In comparison, Rao et al.^5^ utilised systematic sampling to select 64 symptomatic military recruits within a 26-month period.^9,23^

These biases will affect the external validity of the results from this review by potentially overestimating the efficacy of the intervention.^9^ More research is needed to present a conclusive body of evidence to propose a change in clinical practice.

### Recommendations for practice

The evidence review criteria remain inclusive, with all studies being independent to gender, socio-economic status and ethnicity of the participants.^23^ Selection criteria excluded age and potential pathological involvement to maximise the applicability of the collated evidence to the athlete population. Two included studies examined participants with an athletic background.

The clear ultrasound findings of this review alongside the high sensitivity and specificity values concluded in previous literature,^5–7^ indicates the advantageous role of ultrasound as a primary imaging modality in comparison to plain film radiography. The increased probability of diagnosing early cases would ensure early medical intervention and rehabilitation. By ceasing repetitive load upon the affected area, an athlete can ensure a full return to sport without risking long-term implications.^3^ Online Appendix D offers an imaging algorithm proposal for symptomatic athletes. Nonetheless, it is imperative to consider differential diagnoses when identifying optimal primary imaging modalities. Lower limb stress fractures are known to mimic acute osteomyelitis and skeletal malignancies (Ewing’s sarcoma, osteosarcoma, myeloma, and metastatic neuroblastoma), with all periosteal reactions appearing as hyperechoic raised lines on ultrasound.^28,29^ Therefore, radiographic imaging remains crucial to rule out a diagnosis in patients with a suspected stress fracture.

Ultrasound imaging is useful for diagnosing osteomyelitis and skeletal malignancies, can differentiate between acute and chronic infections and tumours and retains diagnostic accuracy in areas complicated by orthopaedic instrumentation.^30^ Additionally, ultrasound has been shown to detect features of osteomyelitis several days earlier than plain film radiography, recognising periosteal elevation by a hypoechoic layer of purulent material and hypoechoic fluid abscesses related to chronic osteomyelitis.^30^ According to Madej et al.,^31^ ultrasound imaging is successful when diagnosing bone tumours, providing a high value in the assessment of musculoskeletal pathologies. They concluded that cortical involvement, pathological separation of the periosteum and periosteal reactions (visualised by hyperechoic reflections) are key features of bone tumours. Similar findings in inflammatory abnormalities were observed, including osteomyelitis and fractures.^31^ Using colour Doppler imaging, ultrasound examines flow direction and velocity within a specified area via intermittent samples of ultrasound waves.^32^ This feature allowed for the visualisation of malignant tissue communicating with bone and assessment of neoplasm vasculature without the need to administer contrast media.^31^ Additionally, just like MRI, ultrasound was able to categorise tumours morphologically by identifying any cystic components, areas of necrosis or haemorrhagic cysts.^31^

An imaging protocol that distinguishes between these differential diagnoses must be developed to reduce the need for radiographic imaging.^28,29^ By identifying key differences between clinical and ultrasonographic presentations, confident diagnoses may be made without risking false negatives. For example, osteomyelitis is typically associated with a fever, and an osteosarcoma is visualised by its ‘sunburst’ appearing periosteal reaction using ultrasonography.^28^

To initiate an interest in research, this systematic review collated secondary data, since it is inexpensive, fast and encompasses a large sample size.^33,34^ Future research regarding appearances of stress fractures using ultrasound for early medical intervention requires the collaboration of primary data alongside this systematic review to assess external validity of the results.^33^ Primary data has full control over study design, so further research should investigate:

1. The accuracy of ultrasound in differentiating between stress fractures, osteomyelitis and skeletal malignancies.
2. Ultrasound sensitivity in diagnosing lower limb stress fractures at early stages.

Future systematic reviews can determine sensitivity and specificity values via a meta-analysis of the primary data. If favourable, these may highlight the benefit of utilising ultrasound as the primary imaging modality, therefore influencing clinical practice as well as potentially reducing the return-to-sport time in symptomatic athletes.

## Conclusion

Our review has outlined the key preliminary image findings for a positive lower limb stress fracture diagnosis using ultrasound, extracted from eight recent publications.

This specification may aid operators when diagnosing athletes with suspected stress fractures and reduce the reliance on plain film radiography. However, due to differential diagnoses, radiographic and MR imaging cannot be omitted from diagnostic imaging until ultrasound is able to reliably exclude pathological involvement. Future research should focus upon identifying differential appearances when utilising ultrasound to confidently identify stress fractures, by combining clinical presentation with preliminary image findings. This way, ultrasound can be the primary imaging modality for detecting lower limb stress fractures in athletes, allowing for universally accessible, on-site imaging.

## Supplemental Material

sj-pdf-1-ult-10.1177_1742271X21995523 - Supplemental material for Preliminary image findings of lower limb stress fractures to aid ultrasonographic diagnoses: A systematic review and narrative synthesis Click here for additional data file. Supplemental material, sj-pdf-1-ult-10.1177_1742271X21995523 for Preliminary image findings of lower limb stress fractures to aid ultrasonographic diagnoses: A systematic review and narrative synthesis by Madeleine Schaper and James Harcus in Ultrasound

sj-pdf-2-ult-10.1177_1742271X21995523 - Supplemental material for Preliminary image findings of lower limb stress fractures to aid ultrasonographic diagnoses: A systematic review and narrative synthesis Click here for additional data file. Supplemental material, sj-pdf-2-ult-10.1177_1742271X21995523 for Preliminary image findings of lower limb stress fractures to aid ultrasonographic diagnoses: A systematic review and narrative synthesis by Madeleine Schaper and James Harcus in Ultrasound

sj-pdf-3-ult-10.1177_1742271X21995523 - Supplemental material for Preliminary image findings of lower limb stress fractures to aid ultrasonographic diagnoses: A systematic review and narrative synthesis Click here for additional data file. Supplemental material, sj-pdf-3-ult-10.1177_1742271X21995523 for Preliminary image findings of lower limb stress fractures to aid ultrasonographic diagnoses: A systematic review and narrative synthesis by Madeleine Schaper and James Harcus in Ultrasound

sj-pdf-4-ult-10.1177_1742271X21995523 - Supplemental material for Preliminary image findings of lower limb stress fractures to aid ultrasonographic diagnoses: A systematic review and narrative synthesis Click here for additional data file. Supplemental material, sj-pdf-4-ult-10.1177_1742271X21995523 for Preliminary image findings of lower limb stress fractures to aid ultrasonographic diagnoses: A systematic review and narrative synthesis by Madeleine Schaper and James Harcus in Ultrasound
