# Supplementary material for: Preliminary image findings of lower limb stress fractures to aid ultrasonographic diagnoses: A systematic review and narrative synthesis
Source: Ultrasound. 2021 Mar 9;29(4):208–17. doi: 10.1177/1742271X21995523 (PMC8579372; doi:10.1177/1742271X21995523)
Supplement: sj-pdf-1-ult-10.1177_1742271X21995523 - Supplemental material for Preliminary image findings of lower limb stress fractures to aid ultrasonographic diagnoses: A systematic review and narrative synthesis [file sj-pdf-1-ult-10.1177_1742271X21995523.pdf]

Appendix A

Collated search results using electronic databases and hand searching

DATABASE AND TIME LIMITS	LANGUAGE	SEARCH DATE	SEARCH TERMS	NUMBER OF HITS	NUMBER OF DUPLICATES REMOVED	NUMBER OF IRRELEVANT ARTICLES	NUMBER OF EXCLUDED WITH REASONS	NUMBER RELEVANT	DUPLICATE REMOVED BETWEEN DATABASES
Ovid Medline 2009 - present	English	03/01/2020	Ultrasound OR ultrasonography Stress fracture* OR Bone stress* Clinical finding* OR presentation OR diagnos* Sensitivity OR specificity	186<br><br>2009-present removed 97	15 removed (171 hits)<br><br>**74** results	60	**12** removed after exclusion criteria applied.<br><br>Studies removed via exclusion criteria: systematic reviews (10), no full text available (2).	2	1 – included in CINAHL search
CINAHL 2009-present	English	03/01/2020	Ultrasound OR ultrasonography Stress fracture* OR Bone stress injury Clinical finding* OR clinical presentation OR diagnosis Sensitivity OR specificity	**83**	Automatic removal	70	**9** removed after critical appraisal<br><br>Exclusion criteria applied: systematic reviews (2), no full text available (3), duplicates within the database search (3)<br>*1 (Drakonaki et al., (35)) – removed post critical appraisal*	4	0
PubMed 2009 - present	English	03/01/2020	(ultrasound) OR ultrasonograph*) AND sensitivity) AND specificity) AND findings) AND presentation) AND diagnosis) AND stress fracture*) AND bone stress	**16**	Automatic removal	14	**2** removed<br><br>Exclusion criteria applied: systematic reviews (2)	0<br>Non relevant	0
Google Scholar 2009-present	English	03/01/2020	Ultrasound Diagnosi* Stress fracture Lower limb	16,900 Only page 1 and 2 viewed: **20** hits. Sorted by best match	N/A	9	**3** removed<br><br>Exclusion criteria applied: systematic reviews (2), pathological involvement (1)	3	4 – previously included in CINAHL (3) and Ovid Medline search (1)
