# Supplementary material for: Preliminary image findings of lower limb stress fractures to aid ultrasonographic diagnoses: A systematic review and narrative synthesis
Source: Ultrasound. 2021 Mar 9;29(4):208–17. doi: 10.1177/1742271X21995523 (PMC8579372; doi:10.1177/1742271X21995523)
Supplement: sj-pdf-2-ult-10.1177_1742271X21995523 - Supplemental material for Preliminary image findings of lower limb stress fractures to aid ultrasonographic diagnoses: A systematic review and narrative synthesis [file sj-pdf-2-ult-10.1177_1742271X21995523.pdf]

Appendix B

An example of the Ovid Medline search (03/01/2020)

# ▲	Searches	Results	Type	Actions	Annotations
1	ultrasound.mp. [mp=ti, ab, hw, tn, ot, dm, mf, dv, kw, fx, dq, nm, kf, ox, px, rx, an, ui, sy, ds, on, tx, sh, ct, bt]	677207	Advanced	Display Results More ▾	
2	ultrasonography.mp. [mp=ti, ab, hw, tn, ot, dm, mf, dv, kw, fx, dq, nm, kf, ox, px, rx, an, ui, sy, ds, on, tx, sh, ct, bt]	409662	Advanced	Display Results More ▾	
3	stress fracture*.mp. [mp=ti, ab, hw, tn, ot, dm, mf, dv, kw, fx, dq, nm, kf, ox, px, rx, an, ui, sy, ds, on, tx, sh, ct, bt]	11996	Advanced	Display Results More ▾	
4	bone stress*.mp. [mp=ti, ab, hw, tn, ot, dm, mf, dv, kw, fx, dq, nm, kf, ox, px, rx, an, ui, sy, ds, on, tx, sh, ct, bt]	4136	Advanced	Display Results More ▾	
5	clinical finding*.mp. [mp=ti, ab, hw, tn, ot, dm, mf, dv, kw, fx, dq, nm, kf, ox, px, rx, an, ui, sy, ds, on, tx, sh, ct, bt]	88902	Advanced	Display Results More ▾	
6	presentation.mp. [mp=ti, ab, hw, tn, ot, dm, mf, dv, kw, fx, dq, nm, kf, ox, px, rx, an, ui, sy, ds, on, tx, sh, ct, bt]	898448	Advanced	Display Results More ▾	
7	sensitivity.mp. [mp=ti, ab, hw, tn, ot, dm, mf, dv, kw, fx, dq, nm, kf, ox, px, rx, an, ui, sy, ds, on, tx, sh, ct, bt]	2296844	Advanced	Display Results More ▾	
8	specificity.mp. [mp=ti, ab, hw, tn, ot, dm, mf, dv, kw, fx, dq, nm, kf, ox, px, rx, an, ui, sy, ds, on, tx, sh, ct, bt]	1624841	Advanced	Display Results More ▾	
9	diagnos*.mp. [mp=ti, ab, hw, tn, ot, dm, mf, dv, kw, fx, dq, nm, kf, ox, px, rx, an, ui, sy, ds, on, tx, sh, ct, bt]	8460426	Advanced	Display Results More ▾	
10	1 or 2	921269	Advanced	Display Results More ▾	
11	3 or 4	15685	Advanced	Display Results More ▾	
12	7 or 8	2971412	Advanced	Display Results More ▾	
13	5 or 6 or 9	8914093	Advanced	Display Results More ▾	
14	10 and 11 and 12 and 13	186	Advanced	Display Results More ▾	
15	remove duplicates from 14	171	Advanced	Display Results More ▾	

Contract

Save Remove Combine with: AND OR Deduplicate
