# Supplementary material for: Preliminary image findings of lower limb stress fractures to aid ultrasonographic diagnoses: A systematic review and narrative synthesis
Source: Ultrasound. 2021 Mar 9;29(4):208–17. doi: 10.1177/1742271X21995523 (PMC8579372; doi:10.1177/1742271X21995523)
Supplement: sj-pdf-3-ult-10.1177_1742271X21995523 - Supplemental material for Preliminary image findings of lower limb stress fractures to aid ultrasonographic diagnoses: A systematic review and narrative synthesis [file sj-pdf-3-ult-10.1177_1742271X21995523.pdf]

Appendix C

The hierarchy of study designs applicable to this systematic review (11).

GRADE	STUDY DESIGN	REFERENCES	EVIDENCE LEVEL	QUALITY	HETEROGENEITY	PRECISION
B+	Controlled, non-randomised, observational study	Banal et al., 2009 (29)<br>Rao et al., 2017 (5)	II	High	Consistent	Precise
C	Observational case reports	Amoako et al., 2017 (28)<br>Hoglund et al., 2011 (30)<br>Bianchi et al., 2014 (31)<br>Battaglia et al., 2013 (32)<br>Khy et al., 2012 (33)<br>Bianchi et al., 2017 (7)	IV	Low	Consistent	Imprecise
