# Supplementary material for: Preliminary image findings of lower limb stress fractures to aid ultrasonographic diagnoses: A systematic review and narrative synthesis
Source: Ultrasound. 2021 Mar 9;29(4):208–17. doi: 10.1177/1742271X21995523 (PMC8579372; doi:10.1177/1742271X21995523)

Appendix D

Proposed imaging algorithm that replaces the need for plain film radiography, as adapted from Wright at al. (6).

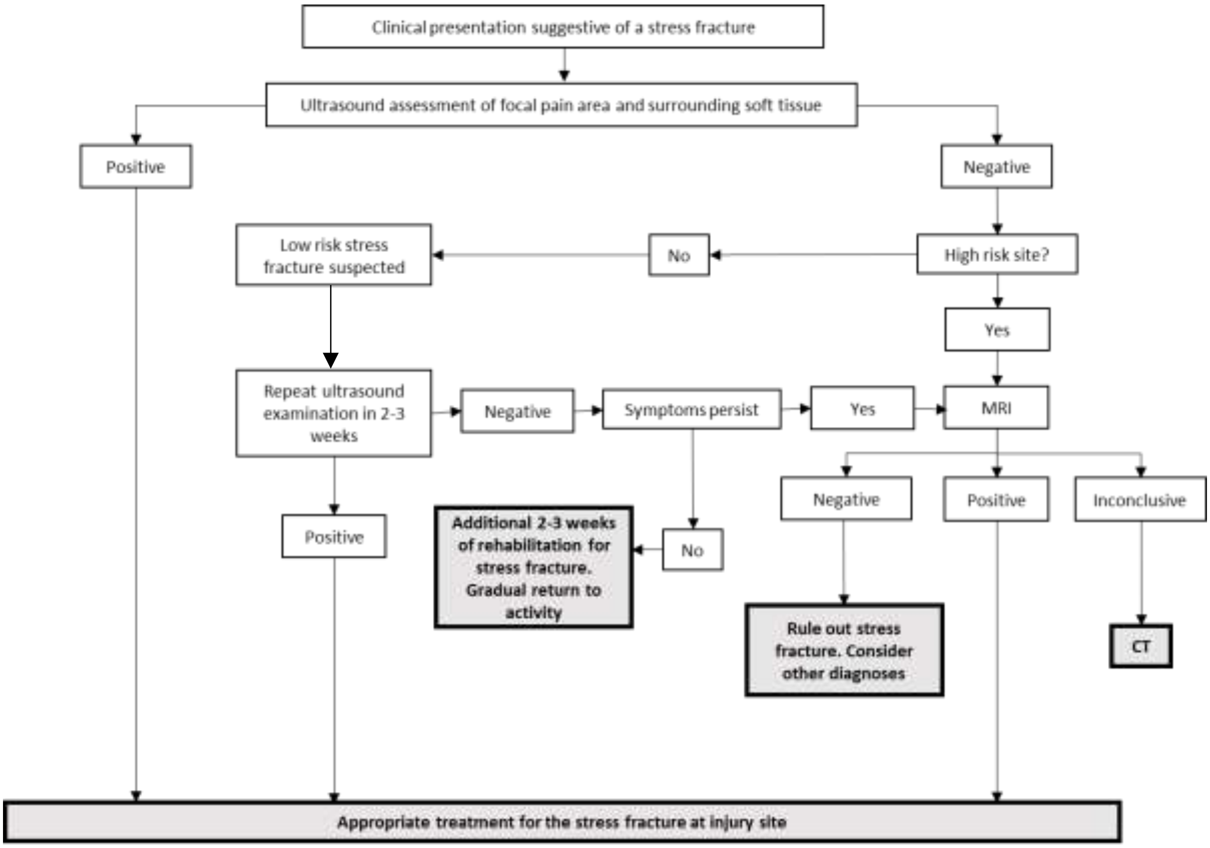

Supplement: sj-pdf-4-ult-10.1177_1742271X21995523 - Supplemental material for Preliminary image findings of lower limb stress fractures to aid ultrasonographic diagnoses: A systematic review and narrative synthesis [file sj-pdf-4-ult-10.1177_1742271X21995523.pdf]
